# Maternal stress and placental function, a study using questionnaires and biomarkers at birth

**DOI:** 10.1371/journal.pone.0207184

**Published:** 2018-11-15

**Authors:** Birthe R. Dahlerup, Emilie L. Egsmose, Volkert Siersma, Erik L. Mortensen, Morten Hedegaard, Lisbeth E. Knudsen, Line Mathiesen

**Affiliations:** 1 Section of Environmental Health, Department of Public Health, University of Copenhagen, Copenhagen, Denmark; 2 The Research Unit for General Practice and Section of General Practice, Department of Public Health, University of Copenhagen, Copenhagen, Denmark; 3 Center for Healthy Aging, University of Copenhagen, Copenhagen, Denmark; 4 Klinik Hedegaard, Copenhagen, Denmark; University of Missouri Columbia, UNITED STATES

## Abstract

**Background:**

Prenatal stress affects the health of the pregnant woman and the fetus. Cortisol blood levels are elevated in pregnancy, and fetal exposure to cortisol is regulated by the placenta enzyme 11β-HSD2. A decrease in enzyme activity allows more maternal cortisol to pass through the placental barrier. Combining the fetal and maternal cortisol to cortisone ratio into the adjusted fetal cortisol exposure (AFCE) represents the activity of the enzyme 11β-HSD2 in the placenta.

**Aim:**

To investigate the effect of prenatal maternal stress on the ratio of cortisol and cortisone in maternal and fetal blood at birth in a normal population.

**Method:**

Maternal self-reported stress was assessed at one time-point, as late in the pregnancy as convenient for the participant, using the Depression Anxiety Stress Scales (DASS-42), Pregnancy Related Anxiety (PRA), and Major Life Events during pregnancy. The study included 273 participants from Copenhagen University Hospital. Maternal and umbilical cord blood was sampled directly after birth and cortisol and cortisone concentrations were quantified using UPLC chromatography. Data were analyzed in a five-step regression model with addition of possible confounders. The primary outcome was AFCE, and plasma concentrations of maternal and fetal cortisol and cortisone were secondary outcomes.

**Results:**

Significant associations were seen for the primary outcome AFCE and the plasma concentrations of maternal cortisol and fetal cortisone with exposure to Pregnancy Related Anxiety (PRA), though the associations were reduced when adjusting for birth related variables, especially delivery mode. The weight of the placenta affected the associations of exposures on AFCE, but not plasma concentrations of cortisol and cortisone in mother and fetus. Moreover, the study demonstrated the importance of delivery mode and birth strain on cortisol levels right after delivery.

**Conclusion:**

Our main finding was associations between PRA and AFCE, which shows the effect of maternal stress on placental cortisol metabolism.

## Introduction

The prevalence of psychosocial stress and anxiety during pregnancy is a cause of concern worldwide, and maternal psychosocial stress has been conceptualized as a teratogen [1]. Maternal and child mental health is addressed in the WHO Mental Health Action Plan (2013–2020), in which it is estimated that about 10% of pregnant women have experienced mental health problems, primarily depression, and this percentage rises to 15.6% when only considering developing countries [2]. A Swedish study found that 20–35% of pregnant women evaluated their own emotional and physical health as poor [3], while 29% of the participating mothers in a survey of a pregnant population from the Copenhagen University Hospital answered “yes” when asked whether they were feeling anxious, stressed or depressed and/or had experienced a traumatizing life event during their pregnancy (unpublished data from LM, n = 48).

Several studies have linked maternal psychosocial stress during pregnancy to a range of effects, both in pregnancy, showing effects on incidence of pre-eclampsia [4] and placental weight [5], in newborn offspring in the infant cortisol response [6] and also later in childhood when studying stress reactivity [7], offspring pediatric disease [8] and asthma and atopic dermatitis [9]. Epidemiological studies of the effects of stress during pregnancy have focused on neurological effects and changes in behavior [6;10;11], and immune system effects such as asthma and allergies [9;12;13].

Elevated cortisol levels in plasma have been used as a biomarker in diagnosis of chronic stress [14]. In pregnant women the plasma concentrations of cortisol are elevated by the feed forward mechanism of the corticotropin-releasing hormone produced by the placenta, and elevated plasma cortisol is therefore a poor biomarker during pregnancy [15;16]. During the first two trimesters of pregnancy, the serum cortisol levels in the fetus are low, except for a peak around gestation week 10 to counteract the effects of hormones from newly formed fetal adrenal tissue. In the third trimester, the fetal serum cortisol levels rise, and is at a maximum at term due to a decline in the placental cortisol metabolizing activity, and the production of cortisol by the fetal adrenal glands [17]. High levels of cortisol during pregnancy have anti-inflammatory and catabolic properties in both the fetus and the pregnant woman.

The mechanisms of maternal psychosocial stress affecting the fetus during pregnancy are assumed to be regulated by placental transfer of hormones, through changes in the expression of placental receptors and enzymes (for reviews, see [18;19]). The placental barrier consists of different cell layers in the human placenta and the placental cell layer most representative of the placental transport and metabolism of hormones is the syncytiotrophoblast. This cell layer expresses the enzyme 11β-HSD2, expressed in tissues that require protection from cortisol, which transforms 80–90% of the maternal cortisol to cortisone passed on to the umbilical and fetal blood [17;20]. The activity of this enzyme has been linked to the effect of maternal psychosocial stress on the offspring, as the metabolic activity of this enzyme protects the fetus from the high maternal cortisol plasma levels (for a review see [21]). The placental gene and mRNA expression of 11β-HSD2, as well as the epigenetic methylation deactivation of 11β-HSD2, has been studied in relation to maternal stress [10;22;23]. The activity of 11β-HSD2 can be studied by comparing cortisol and cortisone concentrations in maternal and fetal blood. Previously, the ratio of maternal cortisol to fetal cortisol and the fetal or maternal cortisol-cortisone ratio have been used as a measure of activity of this enzyme [24–26].

Most studies have been performed in populations of pregnant women who have been diagnosed with depression or have other mental health problems. In contrast, the stress variables in our study are self-reported experiences of depression, anxiety and stress during pregnancy, measured using questionnaires, including selected personality traits of respondents, as these traits can affect the experience of and emotional reactions to stress exposure [27;28]. The aim of the *Maternal Stress and Placental Function* project is to investigate the effect of prenatal maternal psychosocial stress on the adjusted fetal cortisol exposure (AFCE) in a normal pregnant population.

AFCE represents the relative amount of cortisone produced by the placenta measured in fetal blood in relation to how much cortisol the placenta has let pass un-metabolized from maternal blood, corrected for the interindividual differences of these hormone levels in the population–i.e. the placental exposure. The AFCE gives us a measure of the activity of the enzyme 11β-HSD2 until the time of birth. An increase in the AFCE represents a relative increase in fetal cortisol exposure.

## Materials and methods

The current study is a part of the *Maternal Stress and Placental Function* project, conducted in Copenhagen, Denmark. Participants were pregnant women giving birth at Copenhagen University Hospital, where the Department of Obstetrics has around 6000 births a year, of which approximately 22% are Caesarean Sections. Patients admitted to the department are healthy women and women with medical and obstetric complications, as well as women with psychosocial problems. We aimed to investigate the normal population and the criterion for exclusion was age less than 18. The project was approved by the Regional Scientific Ethical Committee of Copenhagen (H-15006254) and the Danish Data Protection Agency (2015-41-4208). All women were informed about the aim of the study and gave written informed consent.

### Recruitment

Recruitment was carried out at four locations connected to the hospital: at the information meetings for all pregnant women; at information meetings for women giving birth to their first child; in the waiting room at the midwives’ offices; and at the information meeting specifically for women giving birth by planned cesarean section. The pregnant women were instructed to answer four written questionnaires in order to measure: 1: relevant personal factors such as socioeconomic status, use of medication, smoking, and alcohol consumption during and before pregnancy, 2: pregnancy-related anxiety, 3: personality, and 4: prevalence of prenatal depression, anxiety and stress. Over a period of 11 months from June 2015 to May 2016, out of 2058 invited families, 562 decided to participate in the study. Only participants with returned questionnaires and successful blood sampling from both mother and umbilical cord directly after birth were included in this study, resulting in 273 participants. Sampling was primarily conducted from Monday to Sunday in the timeframe 7 am to 8 pm.

### Population characteristics

Information regarding lifestyle, BMI, parity, chronic illness, pregnancy complications and medication used during pregnancy was obtained via self-reported questionnaires, and variables connected to the birth and the infant were collected via hospital records. A variable representing the strain of birth (birth strain) was constructed taking into account the length of active labor (vaginal birth 1 point, active labor >12 hours: 2 points, pushing contractions>1 hour: 2 points), augmentation of labor using synthetic oxytocin: (1 point), the use of pain alleviation (non-medical: 1 point or medical: 2 points) and the interventions used during delivery (forceps or vacuum assisted: 1 point or acute section: 2 points). Resulting in a birth strain score of 0 points for elective caesarean section, and a possible score of 1 to 10 points for vaginal births. Placental symmetry was calculated as the measure of the widest place on the placental diameter minus the shortest place on the placental diameter.

### Psychometric measures

Maternal *state* stress was defined as the individual degree of depression, anxiety and stress experienced during the pregnancy, pregnancy- and birth-related thoughts and anxiety, and the experience of major life events during pregnancy. These were assessed using the Depression Anxiety Stress Scales (DASS), Pregnancy Related Anxiety (PRA), and a Major Life Events question. DASS (DASS-42 translated to Danish by Dr Mikael Thastum from the University of Aarhus) contains 42 questions, with depression, anxiety and stress represented by 14 items each [29]. Items were scored 0 to 3 and total scores for each condition were categorized into normal, mild, moderate and severe according to the DASS manual [30]. PRA were assessed using a Danish translation of the 10-item Pregnancy-Related Thoughts (PRT) questionnaire [31] and an additional four items (Birth-Related Thoughts, BRT) (see S1 Table) used by the Copenhagen University Hospital to screen for severely anxious pregnant women. The PRT questions were answered on a four-point Likert scale rating from “not at all (1)” to “very much (4)” and the BRT questionnaire had a five-point scale from 0 to 5. The BRT was transformed and integrated into the PRT with an acceptable internal consistency (α = 0.79), producing a measure of Pregnancy-Related Anxiety (PRA). PRA was categorized into four groups based on total scores in this study: 10–19 defined “no PRA”, 20–25 “some PRA”, 26–31 “moderate PRA” and scores more than 31 “high PRA”. The Major Life Events question, “*have you experienced any major life events during your pregnancy that have led to changes in your state of mind*?”, was answered “yes/no” and defined with examples from the Holmes-Rahe stress inventory of Major Life Events [32].

Maternal *trait* stress was assessed using the Danish version of the NEO-FFI inventory, a well-known measure of the “big five” personality traits of Neuroticism, Extraversion, Openness, Conscientiousness and Agreeableness [33]. The two *traits* of Neuroticism and Conscientiousness were individually included in the analyses as possible confounders. A high score for Neuroticism was defined as the upper 25% of scores (upper quartile) and a low score on Conscientiousness as the lowest 25% of scores (lower quartile).

### Sampling

The participants donated a maternal 20 ml blood-sample and 20–30 ml umbilical cord blood directly after birth. Blood was sampled by venipuncture into 10 ml vacuum tubes containing EDTA, centrifuged for 10 minutes at 4000g without brake and the plasma was collected and stored at -80°C until analysis. All sampling and handling times and times of freezing the samples were noted.

### Analysis of concentration of cortisol and cortisone

The quantification method of cortisol and cortisone is described in detail in the supporting material (S1 File).

In brief: for each analyte, specific isotopically labelled internal standards (IS) (cortisol-d_4_ for cortisol and cortisone-d_8_ for cortisone) were used. The working solution of IS was prepared fresh and consisted of 25 mL 50 mM sodium diphosphate dibasic pentahydrate, pH unadjusted and 1.5 mL of IS mix from Chromsystems. The simplified liquid extraction method was adapted.

Sample analysis was performed using a Waters (Milford, MA, USA) Acquity UPLC system with a Kinetex 2.6 μm EVO C18 column (100Å 100x2.1 mm; Phenomenex, Torrance, CA, USA). Column temperature was 50°C, flow rate was 500 μl/min, and injection volume was 5 μl. The total analysis time was 9 minutes per sample. The mobile phase was a gradient of a mixture of an aqueous mobile phase 0.1% NH_4_OH (v/v) in water (mobile phase A) and an organic phase containing 0.1% NH_4_OH (v/v) in MeOH (mobile phase B).

The passing criteria were defined by measuring the concentration of cortisol and cortisone in QC samples: In each batch, a set of matrix match QC samples were run in order ensure the validity of the batches (low and high). The low control contained cortisol and cortisone at concentrations of 73 nmol/L and 5,5 nmol/L respectively. The high control contained cortisol and cortisone at concentrations of 494 nmol/L and 81,5 nmol/L respectively. If the high controls were within ±15% of the target values and low controls were within ±20% of the target value the batch was considered acceptable. The QC values for the batches that were deemed acceptable the QC values were within ±10% of the target value.

### Outcomes

The primary outcome was cortisol-cortisone ratio between umbilical cord (fetal) blood and maternal blood, referred to as AFCE (adjusted fetal cortisol exposure):

$$AFCE=\frac{Fetal\frac{cortisol}{cortisone}}{Maternal\frac{cortisol}{cortisone}}$$

Maternal and fetal plasma cortisol and cortisone concentrations were included as secondary outcomes.

### Statistical analyses

In order to test for normality in the distribution of cortisol, cortisone and AFCE levels, the Shapiro-Wilk test was performed and yielded a non-normal distribution. Log-transformation was performed and only log fetal cortisol did not obtain normal distribution.

All analyses were performed using linear regression, with stepwise adjustment for confounders and covariates. The associations were assessed in five models. Model 1 was unadjusted, and the other models were adjusted with an increasing selection of potential confounders and intermediate variables: Model 2: personal traits (maternal age, BMI before pregnancy (categorized 1: underweight <18.5kg/m^2^, 2: normal: 18.5–24.9kg/m^2^, 3: overweight: 25–29.9kg/m^2^, and 4: obese: >30kg/m^2^), parity (primipartum) including maternal trait stress (neuroticism (upper quartile) and conscientiousness (lower quartile)), Model 3: lifestyle (smoking during pregnancy, alcohol during pregnancy), Model 4: health (chronic disease, gestational complications (normal birth), asthma medication) and Model 5: delivery (gestational age, gender, mode of delivery (caesarean section or vaginal birth), birth strain, placental weight (log), placenta symmetry (log), time from delivery to maternal blood sample) (see Table 1). The measure of effect is the exponentiated regression coefficient of the exposure– 10^ β–which, because of the log-transform of the outcome, is the factor by which the outcome is multiplied for a unit increase of the exposure.

**Table 1:** Epidemiological model including exposures, potential confounders and outcome variables.

Exposure	Potential confounders	Outcome
*Maternal state stress* *DASS-42*^*a*^ -Depression -Anxiety -Stress PRA^b^ Major life events	Model 2 Personal traits Age BMI Parity *Maternal trait stress*: *NEO-FFI* -neuroticism,conscientiousness	Primary outcome: AFCE^c^ Secondary outcomes: Plasma concentrations of maternal and fetal cortisol and cortisone
	Model 3 Lifestyle Smoking Alcohol	
	Model 4 Health Chronic disease Gestational complications Asthma medication	
	Model 5 Delivery Gestational age Gender Delivery mode Placental weight Placental symmetry Delivery to maternal blood	

^a^DASS-42 is the results from the depression anxiety stress scales.

^b^PRA is the results from the questionnaire on pregnancy related anxiety.

^c^AFCE is the adjusted fetal cortisol response.

ANOVA was used to test the differences in hormone levels and AFCE between vaginal birth and elective caesarean groups, when stratifying the data by birth mode. All statistical modeling and testing was performed in IBM SPSS Statistics 24. Descriptive results are presented categorically with frequency tables and numerically as mean ±SD. A p-value less than 0.05 is deemed statistically significant.

## Results

Descriptive data, lifestyle, self-reported health and birth-related outcomes are shown in Table 2. Caesarean sections were the birth method of 151 (55%) women. Seventy (26%) pregnant women reported having one or more chronic diseases, primarily asthma or allergies and various metabolic, gastrointestinal and dermatological diseases (see Table 2).

**Table 2:** Characteristics of the study population (n = 273).

Variable	Frequency (%)
**Maternal age (in years), mean [range]**	33.6 [23–49]
**Parity, frequency**	
First child	124 (45)
─ twins	6 (2)
Second child or more	149 (55)
**Smoking during pregnancy**	
No	254 (93)
Yes	19 (7)
**Alcohol during pregnancy**	
No	185 (68)
Yes	85 (31)
**BMI before pregnancy, kg/m**^**2**^	
Underweight <18.4	14 (5)
Normal weight 18.5–24.9	193 (71)
Overweight 25–29.9	40 (15)
Obese >30	17 (6)
**Working hours**	
Part time (<30 hours/week)	7 (3)
Full time (≥30 hours/week)	166 (61)
Student	17 (6)
Not working	9 (3)
Changing hours	59 (22)
**Self-reported health**	
**Chronic diseases reported**	
Metabolic (DM type 1 & 2, hypo/hyper-thyroidism, thyroiditis, Graves, PCOS, hepatitis B)	23
Asthma	17
Other immunologic (allergy, hives, immunodeficiency)	11
Skin, joint, bone and connective tissue (Atopic dermatitis, lichen planus, psoriasis, vitiligo, Ehlers Danlos hypermobile, arthritis, endometriosis, adenomyosis, fibromuscular dysplasia, Takayasu arteritis, scoliosis, BRCA1)	16
Gastrointestinal (Morbus Crohn, IBD, Celiac disease, colitis ulceros, anal fissure, hiatus hernia, familial polyposis)	12
Neurological (Depression, Epilepsia, MS relapsing, Impaired vision, papaplegia)	10
Cardiovascular (Stickler syndrome, long QT, Brugada syndrome, Factor V Leiden homo, Prothrombin mutation, cardiolipin antibodies, factor II mutation, Gilbert Meulengracht syndrome)	7
**Gestational complications reported**	
Fertility treatment (IVF, ICSI, Egg-donation)	4
Pregnancy related body burden (Pelvic instability, Hyperemesis, Intrahepatic Cholestasis of pregnancy, GDM)	20
Cardiovascular (Thrombosis, hypertension, arrythmia, arteritis)	11
Gastrointestinal (Obstipation, reflux)	5
Uterus, ovaries, placenta (Pre-ecclampsia+previous, placenta previa, hemorrhaging, Braxton Hicks, ovary cyste, double uterus, previous trakelektomi, abdominal cerclage, swollen labia minor)	16
Fetal (Gemelli, RhD immunization, IUGR+previous, Breech position, premature birth risk, CCAM in fetal lung, fetal thrombocytopenia)	14
Neurological (Stress, anxiety, insomnia)	5
Pain (Pain undefined, pelvic pains, backache, headache, migraine, tooth operation)	10
Infections (UTI, pneumonia, sinus infection, mycoplasma genitalia, abcess, tick bite, influenza, nasal congestion)	18
**Medication during pregnancy (n = 271)**	
No	175 (64)
Yes (Chronic: Loratidin, insulin, methformin, eltroxin, Imurel,Ursochol, Omeprazol, betablocker, Innohep, Setralin, Levetiracetan, Methyldopa, Lutinus, Privigen, Acute: paracetamol, acetyl salicylic acid, ibuprofen, kodein, metadon, Cyclokapron,Promethazin,melatonin, antibiotics)	96 (35)
Asthma medication (Ventoline, Pulmicourt, Salofalk, Oxis turbohaler, Spirocourt, bricanyl, Fuitiform, duoresp)	17
**Gestational age, mean ±SD [range], weeks (n = 260)**	39.4 ±1.2 [36–42]
**Delivery mode**	
Vaginal (including acute Caesarean section)	122 (45)
Caesarean section (elective)	151 (55)
**Birth interventions vaginal births**	
Epidural in vaginal birth	40 (33)
Vacuum or forceps	11 (9)
Acute Caesarean section	7 (6)
**Child gender**	
Male	134 (49)
Female	135 (50)
**Anthropometrics, mean ±SD [range]**	
Birth weight, g	3464 ±487 [2400–5696]
Child length, cm	51.5 ±2.2 [47–59]
Head circumference, cm	35.2 ±1.5 [31–39]
**Placenta weight, mean ±SD [range], g**	712 ±172 [465–1475]
**Apgar score after 5 minutes**	
10	250 (92)
<10	11 (4)
**Sample times, mean ±SD [range], hours**	
Delivery to umbilical cord sample	0:19 ±0:16 [0:01–2:04]
Delivery to maternal blood sample	0:55 ±0:24 [0:05–2:53]
**Outcome values, mean ±SD [range]**	
Maternal cortisol, nmol/L	1276.6 ±528.9 [278.7–3162.0]
Fetal cortisol, nmol/L	118.6 ±111.2 [20.7–585.9]
Maternal cortisone, nmol/L	132.9 ±33.4 [30.4–230.4]
Fetal cortisone, nmol/L	555.5 ±340.1 [116.2–1820.8]
**AFCE** (fetal-maternal cortisol-cortisone ratio)	0.024 ±0.017 [0.00–0.14]

**Fig 1:**
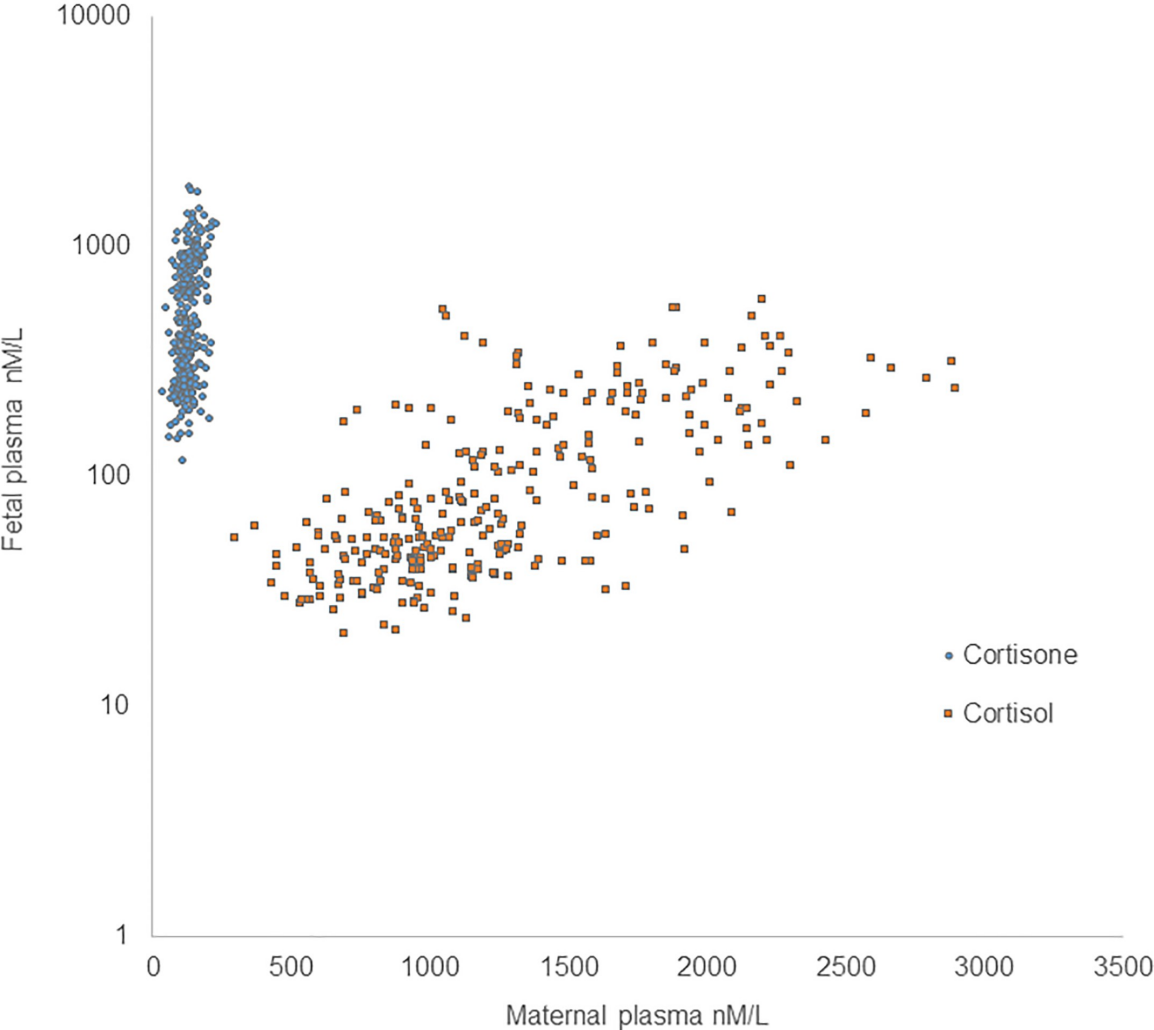
Fetal and maternal cortisone and cortisol. The y-axis is log-scale. Both fetal and maternal plasma cortisone (blue circle) and fetal and maternal cortisol (orange square) values correlate.

Correlations between maternal and fetal levels of cortisol and cortisone are shown in Fig 1.

**Fig 2:**
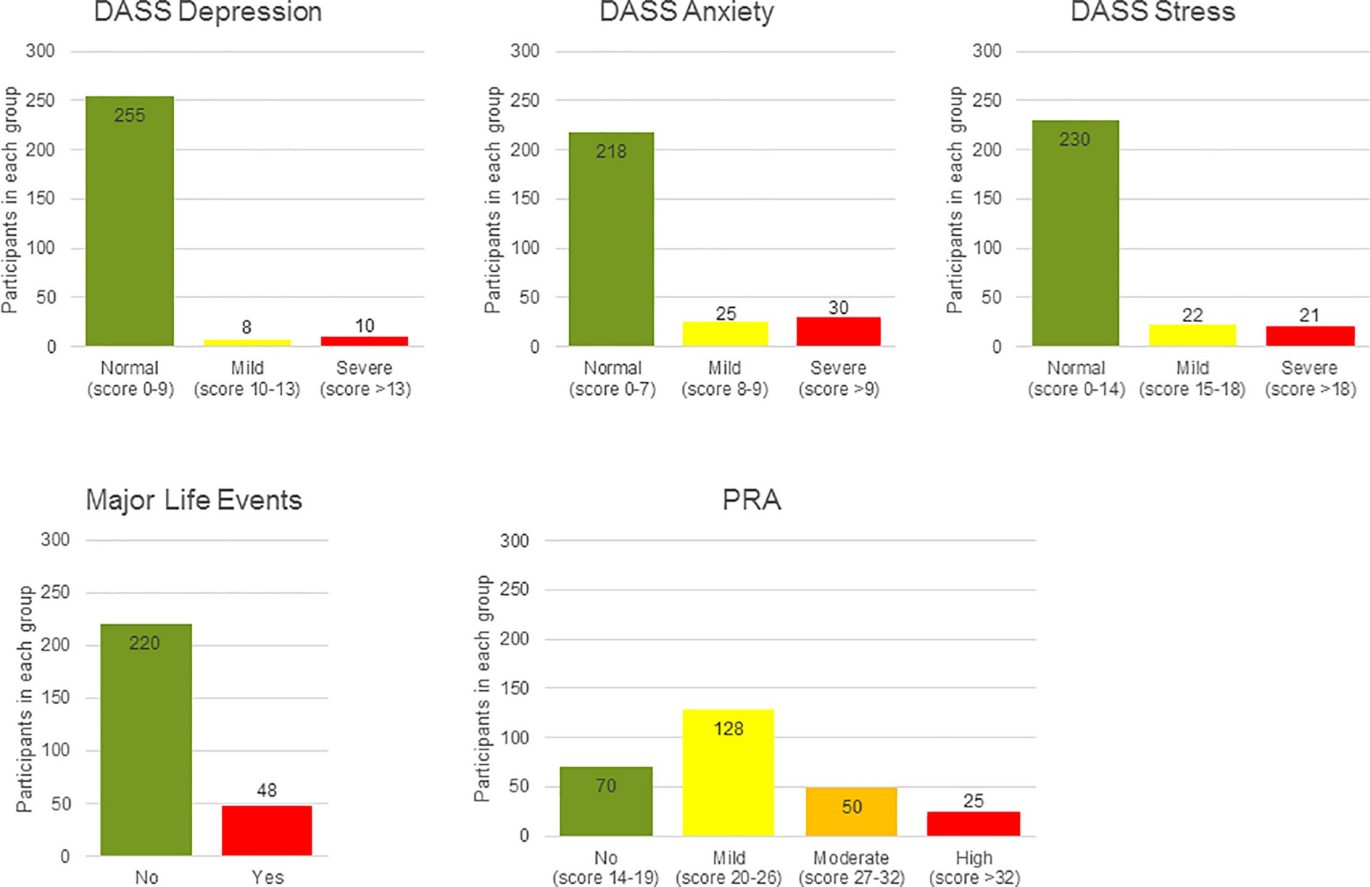
Categorical distribution of the study population based on DASS scores, Major Life Events and PRA. The study population divided into groups according to severeness of maternal (A) Depression, (B) Anxiety and (C) Stress based on the Depression Anxiety and Stress Scales, (D) Major Life events and (E) Pregnancy related anxiety (PRA).

The results from DASS-42, PRA and Major Life Events questionnaires are shown in Fig 2.

### AFCE

Results from the regression analysis (10^β (CI) and p-value for each category of DASS-42, PRA and Major Life Events) are presented in Table 3. Outcomes from the DASS were analyzed as dichotomous “normal” and “increased” due to small group sizes in the “moderate” and “severe” categories (see Fig 2).

**Table 3:** Regression coefficients (10^β), confidence intervals (CI) and levels of significance (p-value) from the five-step regression analysis of state stress exposures and AFCE. The data are presented as 10^β representing the factor by which the mean AFCE is multiplied for each unit increase in exposure.

AFCE	Model 1ᵃ		Model 2ᵃᵇ		Model 3ᵃᵇᶜ		Model 4ᵃᵇᶜᵈ		Model 5ᵃᵇᶜᵈᵉ	
Exposure	10^β (CI)	p	10^β (CI)	p	10^β (CI)	p	10^β (CI)	p	10^β (CI)	p
State-depression	0.789 (0.581–1.040)	0.090	0.783 (0.570–1.549)	0.093	0.774 (0.561–1.035)	0.082	0.757 (0.545–1.019)	0.065	0.787 (0.570–1.054)	0.103
State-anxiety	0.946 (0.805–1.156)	0.694	0.986 (0.818–1.202)	0.927	0.993 (0.820–1.208)	0.963	0.995 (0.820–1.211)	0.973	0.957 (0.802–1.178)	0.773
State-stress	0.877 (0.746–1.109)	0.352	0.875 (0.729–1.130)	0.383	0.867 (0.723–1.125)	0.355	0.865 (0.721–1.125)	0.355	0.910 (0.748–1.167)	0.546
PRA	1.349* (1.007–1.183)	0.032	1.455* (1.023–1.219)	0.013	1.462* (1.026–1.222)	0.012	1.466* (1.023–1.225)	0.013	1.315 (0.991–1.186)	0.080
Major Life Events	1.033 (0.869–1.194)	0.816	1.035 (0.867–1.202)	0.806	1.380 (0.863–1.199)	0.829	1.038 (0.865–1.208)	0.799	1.030 (0.863–1.202)	0.834

^a^ Unadjusted

ᵇ Adjusted for age, BMI, parity, neuroticism upper quartile and conscientiousness lower quartile

ᶜ Adjusted for smoking and alcohol during pregnancy

ᵈ Adjusted for asthma medication, chronic disease and gestational complications

ᵉ Adjusted for gestational age, gender, delivery mode, birth strain, placenta weight and symmetry, and time from delivery to maternal blood samples.

*statistically significant.

A test of correlation between the primary outcome and time from delivery to maternal and fetal blood sample collection was performed using the Pearson Correlation and showed positive correlation between time from delivery to maternal blood sample and log AFCE (r = 0.16, p<0.01). The maternal time variable was therefore included in step 5 of the regression model.

Significant results were seen for the primary outcome AFCE and PRA with approximately 1.5 times increase in AFCE for a unit increase in PRA in models 1 (10^β = 1.349, p = 0.032), and when adjusting for personal traits in model 2 (10^β = 1.455, p = 0.013), lifestyle in model 3 (10^β = 1.462, p = 0.012) and health in model 4 (10^β = 1.466, p = 0.013), but the increase was no longer statistically significant when adjusting for delivery factors in model 5 (10^β = 1.315, p = 0.080) (see Table 3). When stratifying data according to delivery mode, the effect of PRA on AFCE was found primarily in vaginal delivery (model 5: elective Caesarean section: 10^β = 1.102, p = 0.638, vaginal delivery: 10^β = 1.549, p = 0.050) (See S2 Table). No other exposure variable showed any significant effect on AFCE. For all exposure variables (*state*-depression, anxiety and stress, PRA and Major life events) in model 5, the possible confounders showing a significant effect on AFCE were *delivery mode* (10^β = 0.49, p = 0.012, less AFCE in caesarean section), *birth strain* (10^β = 1.77, p = 0.034, more AFCE with higher birth strain) and *placental weight* (10^β = 1.42, p = 0.020, more AFCE in larger placentas) (see S3 Table).

### Plasma concentrations of cortisol and cortisone

As with AFCE, the only exposure variable showing significant effects in models 1–4 was PRA, which was significantly associated with maternal plasma cortisol (10^β = 0.72, p = 0.022 in model 4) and fetal plasma cortisone (10^β = 0.74, p = 0.024 in model 4), but not in model 5 when adjusting for delivery variables.

Like for AFCE, a significant confounder in the exposure effect relationship was *delivery mode* for all exposure variables, in maternal cortisol (10^β~3.3, p<0.000), and fetal cortisol (10^β~3.1, p<0.000) and cortisone (10^β~4.5, p<0.000).

For the two fetal outcomes fetal cortisol and fetal cortisone, there was a significant effect of *parity;* more fetal cortisol (10^β~1.3, p~0.004) and fetal cortisone (10^β~1.3, p~0.004) in first pregnancies. Cortisone in both maternal and fetal plasma was significantly correlated with *maternal age* (10^β~0.6, p~0.001) and (10^β~0.8, p~0.03). In maternal cortisol and fetal cortisone there was an effect of time from delivery to maternal blood sample, with a reverse correlation (10^β~0.6, p<0.000) and (10^β~0.8, p~0.03). The only effect of personality was seen in maternal cortisone, were there was a significant effect of *conscientiousness*, with an increased content of cortisone with increased *conscientiousness* (10^β~1.4, p~0.03).

## Discussion

### Maternal state stress and placental cortisol metabolism

In our study, we saw an association between PRA and our primary outcome measure of placental cortisol metabolism: the AFCE; higher fetal cortisol exposure in the group with the highest scores of pregnancy related anxiety, though the association became non-significant when adjusting for mode of delivery, birth strain and placental weight. Exposure to state-depression, -anxiety or -stress and Major Life Events were not correlated to AFCE, even when adjusting for a range of personal traits, lifestyle, health and birth-related factors.

The association between PRA and AFCE resembles the findings of Glover *et al*. 2009 [24], La Marca-Ghaemmaghami *et al*. 2014 [25], and Hellgren *et al*. 2016 [26], who found effects of maternal state stress on ratios of cortisol and cortisone in maternal and fetal saliva, serum and amniotic fluid. In the study by Glover *et al*., the correlation between maternal and amniotic cortisol was significantly different when the mothers were grouped according to levels of state and trait anxiety. The cortisol levels in mother and amniotic fluid were more similar in more anxious mothers, suggesting lower feto-placental 11β-HSD2 activity [24] La Marca-Ghaemmaghami *et al*. found that maternal salivary responses to the acute stress of amniocentesis, but not self-reported state or trait anxiety, were significantly and positively related to the amniotic fluid cortisone and to amniotic fluid cortisone/(cortisol+cortisone) ratio. This suggested that the acute stress reactivity of the pregnant woman was positively correlated with activity of the placental 11β-HSD2 [25]. Both these studies were performed at gestational week 16–17 and used amniotic fluid as the biomarker for the fetoplacental unit. Hellgren *et al*. studied maternal serum cortisol and cortisone ratio in pregnant women with psychiatric morbidity, determined by self-reported anxiety and depression. The study found a significantly positive correlation between cortisone to cortisol ratio and infant birth weight, driven by maternal psychiatric status [26]. All of these studies included fewer participants than our study but with a potentially higher stress exposure: women with indications for amniocentesis and antenatal psychiatric morbidity, and did not adjust for the covariates of birth method, placenta weight or personality traits. When correcting for delivery mode, and placental weight in model 5, our correlation between PRA and AFCE was no longer statistically significant, although still showing a tendency with a p-value of 0.08. Interestingly, when stratifying the data by delivery mode the correlation between PRA and AFCE in the vaginal delivery group was borderline significant in model 5 with a p-value of 0.05.

When studying the enzyme 11β-HSD2 directly in placental samples, one significant finding relating to fetal cortisol exposure, from a study by Monk *et al*., was the association between DNA-methylation of 11β-HSD2 and maternal stress measured using the Perceived Stress Scales, which in turn was associated with lower fetal coupling [10]. A study by Stroud *et al*. (2016) also found altered placental 11β-HSD2 activity, although with pregnant study participants diagnosed with major depressive disorder. In one-month-old daughters of depressed mothers, salivary cortisol was increased by 50–75% and this effect was mediated by the placental 11β-HSD2-methylation [6]. These methylation studies propose a mechanism by which the activity 11β-HSD2 enzyme is downregulated by prenatal maternal stress. The gene expression of 11β-HSD2 has also been correlated to prenatal maternal anxiety in studies by O’Donnell *et al*. (2011) and Seth *et al*. (2015), demonstrating direct downregulation of gene expression [22;23]. In the study by O’Donnell *et al*., placental samples were donated and state and trait anxiety was reported from pregnant women undergoing caesarean sections, and here a significant negative correlation was seen between 11β-HSD2 gene expression and maternal trait anxiety, with an approximately 30% lower expression in the high-anxiety group. An association was also seen for the state anxiety exposure parameter, but not for self-rated Edinburgh Postnatal Depression Scale (EPDS) scores. However, when looking at enzyme activity in a subgroup of the same study, a significant negative correlation was found for the EPDS score, and a significantly higher enzyme activity in the placenta was found for female fetuses [23]. The EPDS score and the state and trait depression were also found to correlate negatively with 11β-HSD2 gene expression in the study by Seth *et al*., although this was not statistically significant. Here the correlation was particularly prominent during late gestation, as EPDS scores and state anxiety scores showed significant improvement between trimesters [22].

These studies show substantial differences in the exposure and outcome measures in the study of prenatal stress and the effects on the metabolism of cortisol in the placenta. The measures of exposure range from self-reported depression, anxiety and stress using different questionnaires or interviews, to diagnosed major depression disorder, and the outcome measures ranges from gene expression and methylation to different measures of the activity of the enzyme 11β-HSD2. The use of statistical methods and inclusion of covariates also differ among these studies. In our study we have calculated the AFCE, which takes into account both the maternal and fetal cortisone and cortisol, and therefore represents the activity of the placental 11β-HSD2 up until and including the birth. Although there are differences between the mentioned studies, some associations between maternal psychosocial stress during pregnancy and placental 11β-HSD2 activity are found.

### Questionnaire predictivity in a pregnant population

In our study, exposure to PRA showed a significant effect on AFCE, maternal plasma cortisol and fetal plasma cortisone in the first four models and a trend in the adjusted model 5. Data for PRA were obtained using results from questionnaires that were created for a pregnant population and could therefore be sensitive to detecting stressors not found when using questionnaires that are not validated for studies of pregnant women. The data from PRA were also categorized by stress level based on answers from our study population. In the DASS-42 we used the categories given, and most of our population was in the *no* or *low state stress* categories. Differences between scales were also found in other studies. For example, Edinburgh Postnatal Depression Scale results did not correlate with 11β-HSD2 gene expression in the study by O’Donnell *et al*. [23], although *state* and *trait* anxiety measured by other scales showed correlation between the most and least anxious groups.

### Factors affecting the outcome parameters

Mode of delivery, birth strain and placenta weight were the statistically significant factors affecting the correlation of our *state* stress with AFCE. Mode of delivery and birth strain were expected important factors due to the physical strain and pain of vaginal birth, potentially increasing maternal cortisol, as also shown by Stjernholm *et al*. [34]. Indeed, cortisol has been suggested as a biomarker of stress during human term labor [14]. We found that when stratifying our data according to delivery mode, the effect of *state stress* PRA was predominantly seen in the group that gave birth by vaginal delivery; the effect was greater than the effect seen in the unstratified data, and borderline significant (p = 0.050) although the group size was more than halved. All the measured hormones had a significantly higher mean in the vaginal birth group (p<0.000), but the AFCE had a lower mean in the vaginal birth group than the elective Caesarean group (p = 0.016). Therefore, it seems although the hormone levels were higher during vaginal birth, the relative cortisol exposure to the fetus was lower, and the maternal effect of PRA on the placenta was greatest in women delivering by vaginal birth. Delivery mode was also a significant factor in most models comparing the effect of our *state* stress parameters on fetal and maternal cortisol and cortisone. The only outcome for which delivery mode was not a significant factor in the model was maternal cortisone, where maternal age was a significant factor. Cortisone has shown to be age-related caused by increase in 11β-HSD1 enzyme activity with age [35].

The sampling strategy in our study was to take simultaneous (at birth) samples from mother and fetus (umbilical cord) to represent placental cortisol metabolism during pregnancy. The significant effect of delivery mode on our exposure-outcome models demonstrates the importance of including birth method as a covariate when studying the metabolic capacity of the placenta during pregnancy using samples taken directly after birth in both caesarean and vaginal deliveries. Our study population had an overrepresentation of deliveries by caesarean section (55% versus 22% reported by the Department of Obstetrics, Copenhagen University Hospital). This overrepresentation was due to our recruitment strategy, in which the majority of participants were enrolled at meetings for women opting for an elective caesarean section.

Placenta weight is linearly related to the surface area of the villous tissue, a main determinant of the capacity to transport nutrients, as well as maternal plasma levels of placental hormones [36]. The weight of the placenta has, along with other features of placental morphology, been shown to be a more sensitive measure of pregnancy complications, maternal nutritional status, and later health effects on the child than the birth weight [37]. It is therefore reasonable to assume that the size of the placenta also has an influence on the metabolic capacity of the placenta, but in the case of 11β-HSD2 activity, there seems to be no relation to birth weight or placental weight in two studies including 111 and 27 term placentas from normal births [38;39]. In contrast, the size of the placenta had an effect on the correlation between the state stress exposure and the outcome measure of placental cortisol metabolism AFCE in our study. This implies that even though the relative enzyme 11β-HSD2 activity is not increased in larger placentas, there seems to be a mediating effect of size on the placental metabolic capacity.

It is worth noticing that birth strain and placenta weight showed a significant effect on our outcome of AFCE independent of the four measures of maternal and fetal cortisol and cortisone. This shows that the ratio of cortisol and cortisone on fetal and maternal side can be a sensitive proxy measure of placental function, and what factors affect this function.

### Population and method

Our study population is a normal relatively unselected section of the population, and therefore represents normal pregnant women subjected to everyday stressors and pregnancy anxiety. We have not focused on diagnosed psychiatric morbidity, and therefore our results are applicable to the general population. Different diseases as well as medication can affect the cortisol levels of a pregnant woman. We did not see an effect for asthma medication, which indicates that this local use of corticosteroids does not interfere with placental metabolic function.

Out of the initial 2058 persons contacted, the final study included 273 participating mother-placenta-child pairs. In other studies demonstrating the effects of prenatal anxiety, the numbers of participating women were 56 [40], 66 [41] and 262 [24], which suggests that our population size was sufficient to demonstrate differences in exposures and outcomes. A power calculation carried out after the data collection revealed that with our data material we were able to detect differences in AFCE at approximately 13% between normal and increased levels of anxiety, stress, pregnancy-related thoughts and life events with 80% power, which we consider acceptable and relevant. The depression variable that had the smallest group size in the increased group in our study (measured by DASS-42), needed a difference of more than 20% in AFCE between groups before showing sufficient power to detect a statistically significant difference. Our population did not include clinically depressed mothers or mothers with severe mental health issues, making it less likely that we would find critical physiological differences in our population. When planning this study there was a focus on ease of participation, resulting in the decision to sample only at birth and to ask participants to fill in questionnaires at only one point in time during the pregnancy. We believe that this increased the participation rate and aided in recruiting women that would not participate in more time-consuming studies.

We have focused on studies done in humans. When studying reproductive effects and placental transfer it is difficult to extrapolate the effects in animals to effects in humans. This is due to the many differences between pregnancy in the majority of animal and human pregnancies, related to (but not limited to) placental morphology, developmental stage at birth, and child development. The human HPA axis functions differently in pregnancy from most animal models because of the human placental production of corticotropin-releasing hormone and, as shown by Heussner *et al*., there are relevant species differences between the placental steroid metabolisms of humans and rats, which should be considered when attempting to transfer results from rodents to humans [42]. It is therefore not certain that the effects observed in animal experiments, if any, would be similar to those observed in humans.

## Conclusion

Associations between Pregnancy-Related Anxiety (PRA) and adjusted fetal cortisol exposure (AFCE) were seen in our study population of 273 mother-fetus dyads, and these associations were strongest in the vaginal delivery group. The AFCE was significantly associated with placental weight independently of the individual plasma hormone levels, which supports that the AFCE is a measure of placental function. The PRA questionnaires were shown to be more sensitive to the target population than the DASS-42 questionnaire, which demonstrates the importance of validating stress scales to be used in a population of pregnant women.

## Supporting information

S1 Table BRT. Questions about Birth-Related Thoughts, used by Copenhagen University Hospital to screen for anxious pregnant women. Tick a number next to each statement to show how much concern you feel at the moment (only one number for each line).
(DOCX)

S2 Table AFCE stratified by birth mode. Regression coefficients (10^β) and levels of significance (p-value) in the regression analysis of AFCE and state stress exposures stratified by birth mode. Regression coefficients marked *statistically significant, ^#^borderline statistically significant.
(DOCX)

S3 Table AFCE and DASS with possible confounders. Regression coefficients (10^β) and levels of significance (p-value) from step five in the regression analysis of AFCE and state stress exposures including all co-variables.
(DOCX)

S1 File Analysis of cortisol and cortisone. (DOCX)

S1 Dataset Reported data showing maternal age, gestational age, birth weight, and placental weight are grouped with a group size of no less than n = 5. (XLSX)

## Abbreviations

11β-HSD2: 11β-Hydroxysteroid dehydrogenase, a placental enzyme that transforms cortisol into cortisone
BRT: Birth-Related Thoughts, a questionnaire measuring the pregnant woman’s anxiety related to birth
DASS-42: a questionnaire measuring depression, anxiety and stress
AFCE: adjusted fetal cortisol exposure.
NEO-FFI: NEO Five Factor Inventory, a measure of the personality traits of Neuroticism, Extraversion, Openness, Conscientiousness and Agreeableness
PRA: Pregnancy-Related Anxiety, assessed combining PRT and BRT
PRT: Pregnancy-Related Thoughts: a Danish translation of a 10-item questionnaire measuring the pregnant woman’s anxiety related to pregnancy
