# Supplementary material for: Maternal stress and placental function, a study using questionnaires and biomarkers at birth
Source: PLoS One. 2018 Nov 15;13(11):e0207184. doi: 10.1371/journal.pone.0207184 (PMC6237336; doi:10.1371/journal.pone.0207184)
Supplement: S1 Table — Questions about Birth-Related Thoughts, used by Copenhagen University Hospital to screen for anxious pregnant women. Tick a number next to each statement to show how much concern you feel at the moment (only one number for each line). (DOCX) [file pone.0207184.s001.docx]

|  | Not worried |  |  |  |  | Extremely worried |
| --- | --- | --- | --- | --- | --- | --- |
| Going to the hospital | 0 | 1 | 2 | 3 | 4 | 5 |
| Taking maternity leave | 0 | 1 | 2 | 3 | 4 | 5 |
| Whether my partner will be there when I am giving birth | 0 | 1 | 2 | 3 | 4 | 5 |
| The possibility of preterm birth | 0 | 1 | 2 | 3 | 4 | 5 |
