# Supplementary material for: Maternal stress and placental function, a study using questionnaires and biomarkers at birth
Source: PLoS One. 2018 Nov 15;13(11):e0207184. doi: 10.1371/journal.pone.0207184 (PMC6237336; doi:10.1371/journal.pone.0207184)
Supplement: S2 Table — Regression coefficients (10^β) and levels of significance (p-value) in the regression analysis of AFCE and state stress exposures stratified by birth mode. Regression coefficients marked *statistically significant, #borderline statistically significant. (DOCX) [file pone.0207184.s002.docx]

| AFCE | All (n=272) | | Only elective section (n=150) | | Only vaginal births (n=121) | |
| --- | --- | --- | --- | --- | --- | --- |
| *Exposure* | 10^β (CI) | p | 10^β | p | 10^β | p |
| *State-depression* | | | | | | |
| Model 1ᵃ | 0.789 (0.581-1.040) | 0.090 | 0.773 | 0.169 | 0.832 | 0.383 |
| Model 2ᵃᵇ | 0.783 (0.570-1.549) | 0.093 | 0.798 | 0.234 | 0.948 | 0.812 |
| Model 3ᵃᵇᶜ | 0.774 (0.561-1.035) | 0.082 | 0.789 | 0.209 | 0.951 | 0.827 |
| Model 4ᵃᵇᶜᵈ | 0.757 (0.545-1.019) | 0.065 | 0.804 | 0.256 | 0.881 | 0.607 |
| Model 5ᵃᵇᶜᵈᵉ | 0.787 (0.570-1.054) | 0.103 | 0.841 | 0.383 | 1.012 | 0.964 |
| *State-anxiety* | | | | | | |
| Model 1ᵃ | 0.946 (0.805-1.156) | 0.694 | 0.857 | 0.411 | 1.072 | 0.743 |
| Model 2ᵃᵇ | 0.986 (0.818-1.202) | 0.927 | 0.916 | 0.662 | 1.151 | 0.509 |
| Model 3ᵃᵇᶜ | 0.993 (0.820-1.208) | 0.963 | 0.895 | 0.582 | 1.125 | 0.589 |
| Model 4ᵃᵇᶜᵈ | 0.995 (0.820-1.211) | 0.973 | 0.904 | 0.627 | 1.089 | 0.701 |
| Model 5ᵃᵇᶜᵈᵉ | 0.957 (0.802-1.178) | 0.773 | 0.895 | 0.596 | 1.143 | 0.537 |
| *State-stress* | | | | | | |
| Model 1ᵃ | 0.877 (0.746-1.109) | 0.352 | 0.859 | 0.423 | 0.920 | 0.691 |
| Model 2ᵃᵇ | 0.875 (0.729-1.130) | 0.383 | 0.895 | 0.583 | 1.040 | 0.868 |
| Model 3ᵃᵇᶜ | 0.867 (0.723-1.125) | 0.355 | 0.893 | 0.576 | 1.026 | 0.916 |
| Model 4ᵃᵇᶜᵈ | 0.865 (0.721-1.125) | 0.355 | 0.895 | 0.589 | 0.975 | 0.914 |
| Model 5ᵃᵇᶜᵈᵉ | 0.910 (0.748-1.167) | 0.546 | 0.916 | 0.673 | 1.076 | 0.764 |
| *PRA* | | | | | | |
| Model 1ᵃ | **1.349*** (1.007-1.183) | 0.032 | 1.148 | 0.465 | 1.403 | 0.105 |
| Model 2ᵃᵇ | **1.455*** (1.023-1.219) | 0.013 | 1.148 | 0.486 | 1.459 | 0.093 |
| Model 3ᵃᵇᶜ | **1.462*** (1.026-1.222) | 0.012 | 1.114 | 0.593 | 1.476 | 0.087 |
| Model 4ᵃᵇᶜᵈ | **1.466*** (1.023-1.225) | 0.013 | 1.132 | 0.543 | 1.469 | 0.092 |
| Model 5ᵃᵇᶜᵈᵉ | 1.315 (0.991-1.186) | 0.080 | 1.102 | 0.638 | **1.549^#^** | 0.050 |
| *Major Life Events* | | | | | | |
| Model 1ᵃ | 1.033 (0.869-1.194) | 0.816 | 1.127 | 0.527 | 0.904 | 0.629 |
| Model 2ᵃᵇ | 1.035 (0.867-1.202) | 0.806 | 1.213 | 0.315 | 0.991 | 0.963 |
| Model 3ᵃᵇᶜ | 1.380 (0.863-1.199) | 0.829 | 1.172 | 0.421 | 0.986 | 0.949 |
| Model 4ᵃᵇᶜᵈ | 1.038 (0.865-1.208) | 0.799 | 1.233 | 0.297 | 0.942 | 0.782 |
| Model 5ᵃᵇᶜᵈᵉ | 1.030 (0.863-1.202) | 0.834 | 1.334 | 0.158 | 0.845 | 0.434 |
