# Supplementary material for: Maternal stress and placental function, a study using questionnaires and biomarkers at birth
Source: PLoS One. 2018 Nov 15;13(11):e0207184. doi: 10.1371/journal.pone.0207184 (PMC6237336; doi:10.1371/journal.pone.0207184)
Supplement: S3 Table — Regression coefficients (10^β) and levels of significance (p-value) from step five in the regression analysis of AFCE and state stress exposures including all co-variables. (DOCX) [file pone.0207184.s003.docx]

| **AFCE** | DASS-depression | | DASS-anxiety | | DASS-stress | | PRA | | Major Life Events | |
| --- | --- | --- | --- | --- | --- | --- | --- | --- | --- | --- |
|  | 10^β | p | 10^β | p | 10^β | p | 10^β | p | 10^β | p |
| Exposure | 0.79 | 0.103 | 0.96 | 0.773 | 0.91 | 0.546 | 1.32 | 0.080 | 1.03 | 0.834 |
| Maternal age | 1.02 | 0.880 | 1.03 | 0.859 | 1.04 | 0.811 | 1.01 | 0.932 | 1.03 | 0.836 |
| BMI | 0.90 | 0.503 | 0.90 | 0.508 | 0.91 | 0.508 | 0.89 | 0.419 | 0.90 | 0.479 |
| Parity | 1.12 | 0.521 | 1.13 | 0.477 | 1.13 | 0.468 | 1.14 | 0.448 | 1.13 | 0.483 |
| Neuroticism | 1.10 | 0.527 | 1.05 | 0.744 | 1.08 | 0.630 | 0.96 | 0.792 | 1.04 | 0.815 |
| Conscientiousness | 1.27 | 0.111 | 1.25 | 0.140 | 1.26 | 0.128 | 1.28 | 0.102 | 1.26 | 0.131 |
| Smoking | 1.10 | 0.510 | 1.07 | 0.635 | 1.08 | 0.579 | 1.08 | 0.604 | 1.07 | 0.634 |
| Alcohol | 0.91 | 0.537 | 0.92 | 0.586 | 0.93 | 0.614 | 0.95 | 0.698 | 0.92 | 0.594 |
| Asthma medication | 0.97 | 0.852 | 0.98 | 0.900 | 0.97 | 0.839 | 0.97 | 0.819 | 0.98 | 0.880 |
| Chronic disease | 1.14 | 0.408 | 1.14 | 0.409 | 1.14 | 0.409 | 1.14 | 0.388 | 1.14 | 0.393 |
| Gestational compl. | 0,96 | 0.769 | 1.00 | 0.988 | 1.00 | 0.978 | 1.04 | 0.779 | 1.01 | 0.942 |
| Gestational age | 1.02 | 0.899 | 1.03 | 0.890 | 1.02 | 0.901 | 1.05 | 0.777 | 1.03 | 0.872 |
| Gender | 0.97 | 0.803 | 0.96 | 0.786 | 0.95 | 0.755 | 0.98 | 0.888 | 0.97 | 0.825 |
| Delivery mode | **0.50*** | **0.013** | **0.49*** | **0.011** | **0.49*** | **0.012** | **0.53*** | **0.025** | **0.49*** | **0.012** |
| Birth Strain | **1.78*** | **0.030** | **1.78*** | **0.032** | **1.76*** | **0.035** | 1.69 | 0.051 | **1.77*** | **0.034** |
| Placenta weight | **1.41*** | **0.021** | **1.42*** | **0.019** | **1.41*** | **0.021** | **1.39*** | **0.026** | **1.42*** | **0.020** |
| Placenta symmetry | 1.04 | 0.780 | 1.04 | 0.801 | 1.03 | 0.861 | 1.03 | 0.850 | 1.03 | 0.822 |
| Delivery to M blood | 1.29 | 0.076 | 1.30 | 0.075 | 1.29 | 0.082 | 1.31 | 0.066 | 1.29 | 0.076 |
