# Supplementary material for: Maternal stress and placental function, a study using questionnaires and biomarkers at birth
Source: PLoS One. 2018 Nov 15;13(11):e0207184. doi: 10.1371/journal.pone.0207184 (PMC6237336; doi:10.1371/journal.pone.0207184)
Supplement: S1 File — (DOCX) [file pone.0207184.s004.docx]

## **Analysis of Cortisol and Cortisone**

### **Chemicals and reagents**

HPLC grade acetonitrile, ammonium hydroxide, ethyl acetate, sodium diphosphate pentahydrate and isopropanol were from Sigma-Aldrich. Methanol was purchased from Fisher Scientific and formic acid was obtained from Merck. All other chemicals and solvents were of highest analytical grade available.

Novum SLE MAX Simplified Liquid Extraction (SLE) 96-well plates were obtained from Phenomenex; 1.1 mL 96-well deep well plates (Axygen P-DW-11-C-S 96 Well Clear Round Bottom 1.1 mL Polypropylene Deep Well Plate) were obtained from Agilent. The pierceable cover mats were obtained from Fisher Scientific.

For calibration and quality control the 6Plus1® Multilevel Serum Calibrator Set MassChrom Steroid Panel 1 calibration kit MassCheck Steroid Panel 1 Serum Control of Chromsystems (Chromsystems, Gräfelfing, Germany) was used. Internal Standard Mix MassChrom Steroids in serum/plasma were also provided by Chromsystems.

### **Calibration standard, internal standards and quality controls**

Calibration was performed using a lyophilized multilevel serum calibrator set of known increasing concentrations (n=6) of cortisol and cortisone and a blank sample. The concentrations of calibrators were as follows: 30.7, 60.3, 119, 229, 433 and 865 nM/L; 2.67, 6.74, 13.4, 28.9, 59 and 118 nM/L for cortisol and cortisone respectively. Low- and high-concentration lyophilized sera samples were used as quality controls. All calibrators and controls were reconstituted according to the manufacturer’s instructions.

For each analyte, specific isotopically labelled internal standards (cortisol-d_4_ for cortisol and cortisone-d_8_ for cortisone) were used. The working solution of IS was prepared fresh and consisted of 25mL 50mM sodium diphosphate dibasic pentahydrate, pH unadjusted and 1.5 mL of IS mix from Chromsystems.

### **Sample preparation**

The simplified liquid extraction method was adapted. Briefly, 150 μL of patient sample, calibrator or control was transferred in a 1.1 mL 96-well deep well plate (Axygen P-DW-11-C-S 96 Well Clear Round Bottom 1.1 mL Polypropylene Deep Well Plate). After dispensing of 250 μL of internal standard mix, the mixture was shaken mechanically for 30 min at 450 rpm on the orbital shaker.

Then the mixture was transferred into a SLE 96-well plate (Novum SLE MAX plate) and a short and gentle pulse vacuum was applied (~5”of Hg) for 5-10 seconds or until the sample had completely entered the media. After 5 minutes, the SLE plate was placed on the fresh 1.1 mL 96-well plate (Axygen) in the collecting position and 900 μL of ethyl acetate was added to each well. Then the eluent was allowed to pass through the SLE sorbent by gravity flow. After 5 minutes, the extract was evaporated to complete dryness under a slow stream of N_2_ at 40^o^C. The dry residue was reconstituted in 150 μL of initial mobile phase. The collection plate was covered with a pierceable cover and agitated at 450 RPM for 30 minutes prior to LC-MS/MS analysis.

Table 1. Gradient method used for the mobile phase in separation of samples for the LC-MS/MS method

| Step | Start time (min) | %A | %B |
| --- | --- | --- | --- |
| 1 | 0.00 | 80 | 20 |
| 2 | 0.50 | 80 | 20 |
| 3 | 7.00 | 25 | 75 |
| 4 | 7.10 | 0 | 100 |
| 5 | 8.00 | 0 | 100 |
| 6 | 8.10 | 80 | 20 |
| 7 | 9.00 | 80 | 20 |

### **LC-MS/MS conditions**

Sample analysis was performed using a Waters (Milford, MA) Acquity UPLC system with a Kinetex 2.6 μm EVO C18 column (100Å 100x2.1 mm; Phenomenex, Torrance, CA, USA). Column temperature was 50°C, flow rate was 500 µl/min, and injection volume was 5 µl. The total analysis time was 9 minutes per sample. The mobile phase was a gradient of a mixture of an aqueous mobile phase 0.1% NH_4_OH (v/v) in water (mobile phase A) and an organic phase containing 0.1% NH_4_OH (v/v) in MeOH (mobile phase B). The gradient profile is presented in Table 1. Two kinds of wash solution were used between injections (wash solution 1: 0.1% FA in AcN/IPA/MeOH/H2O 25/25/25/25 [v/v]; wash solution 2: 0.1% NH_4_OH [v/v] in 10% MeOH).

A Waters Xevo TQ-S triple quadrupole mass spectrometer (Waters) was operated using the following settings: capillary voltage, 0.60 kV, cone voltage (30-38), collision energy (26-40), depending on analytes; and collision gas, argon at 2.8 x 10^3^ mbar. The mass spectrometer was operating under positive ionization mode and detection of the analytes was based on multiple reaction monitoring (MRM). The mass parameters for both analyte and the internal standards are presented in Table 2. MRM transitions for the analytes and internal standards were collected over the appropriate retention time. The MassLynx software ver. 4.1 (Waters) was used for peak area integration and data analysis.

Table 2. Multiple reaction monitoring: transitions monitored and compound-specific settings.

| Compound | Mass transition (m/z) | Cone voltage | Collision energy |
| --- | --- | --- | --- |
| Cortisol | 363.2→91.1 | 30 | 40 |
| Cortisone | 361.2→105 | 38 | 40 |
| Cortisol-d4 | 667.2→97.1 | 30 | 40 |
| Cortisone-d8 | 369.2→168.1 | 38 | 26 |

The 6-point calibration curves for both cortisol and cortisone were generated by plotting the peak areas of each analyte, corrected by the corresponding internal standard, versus the concentration of the analyte and processed by weighed (1/x) least squares linear regression. The linearity was considered acceptable if the coefficient of correlation was at least 0.98.

The passing criteria were defined by measuring the concentration of cortisol and cortisone in QC samples: In each batch, a set of matrix match QC samples were run in order ensure the validity of the batches (low and high). The low control contained cortisol and cortisone at concentrations of 73 nmol/L and 5,5 nmol/L respectively. The high control contained cortisol and cortisone at concentrations of 494 nmol/L and 81,5 nmol/L respectively. If the high controls were within ±15% of the target values and low controls were within ±20% of the target value the batch was considered acceptable. The QC values for the batches that were deemed acceptable the QC values were within ±10% of the target value.
